# Supplementary material for: Rap2 and TNIK control Plexin-dependent tiled synaptic innervation in C. elegans
Source: eLife. 2018 Jul 31;7:e38801. doi: 10.7554/eLife.38801 (PMC6067881; doi:10.7554/eLife.38801)
Supplement: Supplementary file 1. [file elife-38801-supp1.docx]

**Supplemental Experimental Procedures**

**RT-qPCR**

Total RNA was prepared from the mixed stage of wild type and *rap-2* CRISPR mutants animals using GeneJET RNA purification kit (Thermo Fisher Scientific). RT-qPCR was conducted using Luna® Universal One-Step RT-qPCR Kit (NEB) and CFX384 Touch™ Real-Time PCR Detection System (Bio-Rad) according to the manufacturer’s instructions. *cdc-42* was used as an internal reference.

**List of primers**

genotyping

*plx-1(nc36):*

Forward: CTTCGAGAGCCCCCTCATTCTTGATG

Reverse: CCGGCACACGTTAAACTAGTGCTACCG

*rap-2(gk11)*:

Forward TCTCATCTCCATCGTCGTTCCTGC;

wild type Reverse GAGGGAGTTCAAAGTGGTCGTTC;

mutant Reverse TCCATTCACTGAATGTTCCGC

*rap-2(miz16)* PCR products from *miz16* allele can be digested with *Bam*HI.

Forward: TGATTTTTCGACCGTTGTGGCTC

Reverse: GCCGAAAAACACATAGAATCCCC

*rap-2(miz17 - 20)*

wildtype Forward: AAGTGGTCGTTCTGGGTAGT

mutant Forward: AAGTCGTGGTTCTTGGTTCA

Reverse: GGCTCGAATTTACCAGATTTTACG

*rap-3 (gk3975)*:

Forward: CTTGTTAACTTCAGGTTCCACTGGG

Reverse: GTTCTGGTTGAGCCTTGCACTAGTC

*jnk-1 (gk7)*

Forward: TATCGAGTCCGTTGGGAATGTGAG

wild type Reverse: GTTCCATGAGAATTCCTCCTCCTG

mutant Reverse: GCCCGATAGTATCTTGTCACAACG

plasmid construction

*rap-1* promoter (cloned into *Sph*I/*Asc*I sites of the pSM vector)

Forward: GGGCATGCCAATTCTCAATCATTAGTTTTCGGG

Reverse: GGGGCGCGCCTCTTTTTTTGAAGATCTGTTATGGTGT

*rap-2* promoter (cloned into *Sph*I/*Asc*I sites of the pSM vector)

Forward: GGGGCATGCGTGTGATCTCCGGAGCAATTTG

Reverse: GGGGGCGCGCCTGAGAGTTTTTTGCTGAAAATC

*rap-3* promoter (cloned into *Sph*I/*Asc*I sites of the pSM vector)

Forward: GGGGCATGCCGCTCTAGTACCATCTTTCC

Reverse: GGGGGCGCGCCCTCTTTCATTTCTTTTTGTATC

*rap-1* cDNA (cloned into *Asc*I/*Kpn*I sites of the ΔpSM vector)

Forward: GGGGGCGCGCCATGCGGGAGTATAAGATTGTTGTGC

Reverse: GGGGGTACCTCACATGATGACACACGAGCAGCACTG

*rap-2* cDNA (cloned into *Asc*I/*Kpn*I sites of the ΔpSM vector)

Forward: GGGGGCGCGCCATGAGGGAGTTCAAAGTGGTCG

Reverse: GGGGGTACCTCACATCAGAGAGCAACATGATTTG

*rap-2* G12V mutation

Forward: TTCTGGGTAGTGTTGGTGTCGGAAAA

Reverse: TTTTCCGACACCAACACTACCCAGAA

*rap-2* S17A mutation

Forward: GGTGTCGGAAAAGCCGCGTTGACGGTGCA

Reverse: TGCACCGTCAACGCGGCTTTTCCGACACC

*rap-2* repair template (cloned into *Eco*RI site of the pBluescriptII SK+ vector)

Forward: CGATAAGCTTGATATCGAAAGCTACGATCGCTCGTGTAC

Reverse: GATCCCCCGGGCTGCAGGAACGGCCAATTTGTCCCATTCC

*mig-15* genomic DNA (cloned into *Asc*I/*Kpn*I sites of the ΔpSM vector)

Forward: GGGGGCGCGCCATGTCGTCATCAGGACTCGACGAGATTGA

Reverse: GGGGGTACCTTACCAATTTGTCAACCCTGGCTTATTTA

*hRap2a* cDNA (cloned into *Asc*I/*Kpn*I sites of the pSM vector)

Forward: GGGGGCGCGCCATGCGCGAGTACAAAGTGGTGGTG

Reverse: GGGGGTACCCTATTGTATGTTACATGCAGAACAG

human Rap2a cDNA (cloned into *Xho*I/*Bam*HI sites of pCI-eGFP-Rap1 plasmid to replace Rap1 with Rap2a)

wildtype Forward: GATCTCGAGGGATGCGCGAGTACAAAGTGGTGGTGCTGGGCTC

G12V Forward: ATCTCGAGGGATGCGCGAGTACAAAGTGGTGGTGCTGGGCTCGGTCGGGG

S17A Forward: ATCTCGAGGGATGCGCGAGTACAAAGTGGTGGTGCTGGGCTCGGGCGGGGTAGGCAAAGCCG

Reverse: GGTGGATCCACCTCCGGAGCCTTTGTCAGGCTG

eGFP-Rap2a (cloned into *Asc*I/*Kpn*I sites of the ΔpSM vector with *mig-13* promoter)

Forward: ATTCAGAATTTCAGGTAGGCGCGCCATGGTGAGCAAGGGCGAGGA

Reverse: CTCAGATATCAATACCATGGTACCCTATTGTATGTTACATGCAGAAC

CRISPR

*rap-2* sgRNA

Forward: GTCGGAAAATGTTTTAGAGCTAGAAATAGCAAG

Reverse: ACCTCCACTACAAACATTTAGATTTGCAATTCAATTATATAG

*rap-2* repair template (cloned into *Eco*RI site of the pBluescriptII SK+ vector)

5' fragment and 3'fragment were amplified separately and cloned simultaneously into the pBluescript SK(+) using SLiCE method.

5' Forward: CGATAAGCTTGATATCGAAAGCTACGATCGCTCGTGTAC

5' Reverse: CTGAACCAAGAACCACGACTTTGAACTCCCTCATTGAGAG

3' G12V Forward: AGTCGTGGTTCTTGGTTCAGTTGGTGTCGGAAAATCGGCG

3' G12V Forward: AGTCGTGGTTCTTGGTTCAGGAGGTGTCGGAAAAGCGGCG

3' Reverse: GATCCCCCGGGCTGCAGGAACGGCCAATTTGTCCCATTCC

*rap-2* sequencing (1867bp)

Forward: GTCCTGCGCCCTTCTTTGTTCTG

Reverse: GGCTCGAATTTACCAGATTTTACG

RT-qPCR

*rap-2*

Forward: CGTTGACGGTGCAATTTGTCAG

Reverse: CCTGCAGTCTCCAGAATTTCCAC

*cdc-42*

Forward: CTGCTGGACAGGAAGATTACG

Reverse: CTCGGACATTCTCGAATGAAG

**Repair template sequence**

***rap-2 G12V (miz16)***

TAAAGATAGCTGCCCAAAAAATTTGCTACTCCACTTTAGAATCAATTTCAGCCTGAAAACTTTTCAAATCCGAGGTTTTAATTTCAGTTTTCCTATTACTTTTCAACATCAAATTGCCGAGAATGTTATAGCAATAATGAAATGAAAAAAGGAAGACGAACGAACGAGAAACAACAAAAAAACTTTCACAATTTCCATTTCATTCATACCATCATCATCATCTCTTCAAACTTCCGTTTCATTCTTTCCCATTTTTTCCGGTTATTGTTGTCTGTGTGTGTGTGGTGATGTAATAGTTCTACTACAATAATTCGTTCTTCTTCTTTTCTCACTTTTCCCACCACATTCACAATCACACCTAATCCAATTTATTGATTTTCAGCAAAAAACTCTCAATGAGGGAGTTCAAAGTGGTCGTTCTGGGATCCGTTGGTGTCGGAAAATCGGCGTTGACGGTGCAATTTGTCAGTAGCACATTCATCGAGAAGTATGATCCGACGATTGAGGACTTTTATCGCAAGGAAATTGAAGTGAGAGCGATTCATTTTATTTTAAATTTAAAAAAAAAAAAAGAAAA

***rap-2 G12V (miz17, miz18)***

AGCTACGATCGCTCGTGTACTCCCCGAGGAGAAGGAGTTTACAGTTTTTCGTTAAAATGCTCGATTTTTGACTTTTTTTGCAAATTCGTTCGGTATTCAACAGATTTTCTCTTTTTTCCAGTTTTTTACAAGAAAACTGATGATTTTTTGATTAAAAACTACTGAAACCGATAATTTTTAGACTTTCGCCGAAAAACACATAGAATCCCCAAAAATCCTATAAACAAGAACTTTTATAGCTTTAAATACATGAAGAAGTAGTAAAAATGCTACAAAATCAAATATTGTAGGAAAAAACTGTAAAATTCTTTTTCTCAGGGAGCAAGTAATGGGAGTTCTAAAATCTTTCAATTTCAAAGCTTAATATAATGTTTTAATGAATTTTTAGAAACTTTAAACCATTCTGAAAATTTCAAGCGCCATGATTTCAACCGAATTTTCTTTTTTTTTTTTTAAATTTAAAATAAAATGAATCGCTCTCACTTCAATTTCCTTGCGATAAAAGTCCTCAATCGTCGGATCATACTTCTCGATGAATGTGCTACTGACAAATTGCACCGTCAACGCCGATTTTCCGACACCAACTGAACCAAGAACCACGACTTTGAACTCCCTCATTGAGAGTTTTTTGCTGAAAATCAATAAATTGGATTAGGTGTGATTGTGAATGTGGTGGGAAAAGTGAGAAAAGAAGAAGAACGAATTATTGTAGTAGAACTATTACATCACCACACACACACAGACAACAATAACCGGAAAAAATGGGAAAGAATGAAACGGAAGTTTGAAGAGATGATGATGATGGTATGAATGAAATGGAAATTGTGAAAGTTTTTTTGTTGTTTCTCGTTCGTTCGTCTTCCTTTTTTCATTTCATTATTGCTATAACATTCTCGGCAATTTGATGTTGAAAAGTAATAGGAAAACTGAAATTAAAACCTCGGATTTGAAAAGTTTTCAGGCTGAAATTGATTCTAAAGTGGAGTAGCAAATTTTTTGGGCAGCTATCTTTAGCTGAAATGTTCAAATTTGCCAACCTCTCAGAGCCACAACGGTCGAAAAATCAATATTTATGTATTAAAGCTCCTTTGTTTTGCAAATTTCGGTCGTTTATATCTAATTTTTTGATAATTTAGGCACATTTTCAGTCAATAGGTGTACAAACTAACAGAAAAATAATGTAAAAACTTTAGAAATCCTCTAAACGAGACAGTAATTGGATAAATATTAATTCCGGCCGCTAGGAATGGGACAAATTGGCCG

***rap-2 G12V (miz19, miz20)***

AGCTACGATCGCTCGTGTACTCCCCGAGGAGAAGGAGTTTACAGTTTTTCGTTAAAATGCTCGATTTTTGACTTTTTTTGCAAATTCGTTCGGTATTCAACAGATTTTCTCTTTTTTCCAGTTTTTTACAAGAAAACTGATGATTTTTTGATTAAAAACTACTGAAACCGATAATTTTTAGACTTTCGCCGAAAAACACATAGAATCCCCAAAAATCCTATAAACAAGAACTTTTATAGCTTTAAATACATGAAGAAGTAGTAAAAATGCTACAAAATCAAATATTGTAGGAAAAAACTGTAAAATTCTTTTTCTCAGGGAGCAAGTAATGGGAGTTCTAAAATCTTTCAATTTCAAAGCTTAATATAATGTTTTAATGAATTTTTAGAAACTTTAAACCATTCTGAAAATTTCAAGCGCCATGATTTCAACCGAATTTTCTTTTTTTTTTTTTAAATTTAAAATAAAATGAATCGCTCTCACTTCAATTTCCTTGCGATAAAAGTCCTCAATCGTCGGATCATACTTCTCGATGAATGTGCTACTGACAAATTGCACCGTCAACGCCGCTTTTCCGACACCTCCTGAACCAAGAACCACGACTTTGAACTCCCTCATTGAGAGTTTTTTGCTGAAAATCAATAAATTGGATTAGGTGTGATTGTGAATGTGGTGGGAAAAGTGAGAAAAGAAGAAGAACGAATTATTGTAGTAGAACTATTACATCACCACACACACACAGACAACAATAACCGGAAAAAATGGGAAAGAATGAAACGGAAGTTTGAAGAGATGATGATGATGGTATGAATGAAATGGAAATTGTGAAAGTTTTTTTGTTGTTTCTCGTTCGTTCGTCTTCCTTTTTTCATTTCATTATTGCTATAACATTCTCGGCAATTTGATGTTGAAAAGTAATAGGAAAACTGAAATTAAAACCTCGGATTTGAAAAGTTTTCAGGCTGAAATTGATTCTAAAGTGGAGTAGCAAATTTTTTGGGCAGCTATCTTTAGCTGAAATGTTCAAATTTGCCAACCTCTCAGAGCCACAACGGTCGAAAAATCAATATTTATGTATTAAAGCTCCTTTGTTTTGCAAATTTCGGTCGTTTATATCTAATTTTTTGATAATTTAGGCACATTTTCAGTCAATAGGTGTACAAACTAACAGAAAAATAATGTAAAAACTTTAGAAATCCTCTAAACGAGACAGTAATTGGATAAATATTAATTCCGGCCGCTAGGAATGGGACAAATTGGCCG
